# Causal reasoning with mental models

**DOI:** 10.3389/fnhum.2014.00849

**Published:** 2014-10-28

**Authors:** Sangeet S. Khemlani, Aron K. Barbey, Philip N. Johnson-Laird

**Affiliations:** ^1^Navy Center for Applied Research in Artificial Intelligence, Naval Research LaboratoryWashington, DC, USA; ^2^Beckman Institute for Advanced Science and Technology, University of Illinoi at Urbana-ChampaignUrbana, IL, USA; ^3^Department of Psychology, Princeton UniversityPrinceton, NJ, USA; ^4^Department of Psychology, New York UniversityNew York, NY, USA

**Keywords:** causal reasoning, mental models, explanations, enabling conditions, lateral prefrontal cortex

## Abstract

This paper outlines the model-based theory of causal reasoning. It postulates that the core meanings of causal assertions are deterministic and refer to temporally-ordered sets of possibilities: *A causes B to occur* means that given *A*, *B* occurs, whereas *A enables B to occur* means that given *A*, it is possible for *B* to occur. The paper shows how mental models represent such assertions, and how these models underlie deductive, inductive, and abductive reasoning yielding explanations. It reviews evidence both to corroborate the theory and to account for phenomena sometimes taken to be incompatible with it. Finally, it reviews neuroscience evidence indicating that mental models for causal inference are implemented within lateral prefrontal cortex.

## Introduction

All reasonings concerning matter of fact seem to be founded on the relation of Cause and Effect.

David Hume (1748/1988)

In *An Enemy of the People*, the protagonist, Dr. Stockmann, discovers that waste runoff from the town tanneries is contaminating the water supply at the public baths, a municipal project that he himself has led with his brother, the mayor. He exclaims:

“The whole Bath establishment is a whited, poisoned sepulcher, I tell you—the gravest possible danger to the public health! All the nastiness up at Molledal, all that stinking filth, is infecting the water in the conduit-pipes leading to the reservoir; and the same cursed, filthy poison oozes out on the shore too… ” (Act I, An Enemy of the People)

Dr. Stockmann acts on his conviction by alerting the mayor to the threat of contamination—and suffers as a result. His actions are based on his causal beliefs:

- The waste from the tanneries causes contamination in the baths.
- The townspeople are going to allow tourists at the baths to be at risk.
- It is necessary to try to prevent further contamination.

Ibsen's play examines how these beliefs and Stockmann's consequent actions lead him to become a pariah—an enemy of the people—much as Ibsen perceived himself to be, as a result of his revealing depictions of Norwegian society.

Our research is more prosaic: it examines how individuals interpret and represent causal relations, how they reason from them and use them in explanations, and how these mechanisms are implemented in the brain. This paper brings together these various parts in order to present a unified theory of causal reasoning in which mental models play a central role. The theory of mental models—the “model theory,” for short—ranges over various sorts of reasoning—deductive, inductive, and abductive, and it applies to causal reasoning and to the creation of causal explanations.

The organization of the paper is straightforward. It begins with a defense of a deterministic theory of the meaning of causal assertions. It explains how mental models represent the meanings of causal assertions. It shows how the model theory provides a framework for an account of causal reasoning at three levels of analysis (Marr, 1982): what the mind computes, how it carries out these computations, and how the relevant mechanisms are realized in the brain, that is, the functional neuroanatomy of the brain mechanisms underlying causal reasoning.

## The meaning of causal relations

One billiard ball strikes another, which moves off at speed. If the timing is right, we see a causal relation even when the billiard balls are mere simulacra (Michotte, 1946/1963). Many causal relations, however, cannot be perceived, and so the nature of causation is puzzling. Indo-European languages, such as English, contain many verbs that embody causation. They are highly prevalent because, as Miller and Johnson-Laird (1976) argued, causation is an operator that, like time, space, and intention, occurs in verbs across all semantic domains. Each of the verbs in the following sentences, for example, embodies the notion of cause and effect:

The wind pushed the fence down (caused it to fall down).

His memory of his behavior embarrassed him (caused him to feel embarrassed).

She showed the ring to her friends (caused it to be visible to them).

Scholars in many disciplines have studied causation, but they disagree about its philosophical foundations, about its meaning, and about causal reasoning. For Hume (1748/1988), causation was an observed regularity between the occurrence of the cause and the occurrence of the effect. As he wrote (p. 115): “We may define a cause to be an object followed by another, and where all the objects, similar to the first, are followed by objects similar to the second.” For Kant (1781/1934), however, a necessary connection held between cause and effect, and he took this component to be a part of an innate conception of causality. What is common to both views is that causal relations are, not probabilistic, but deterministic, and the same claim is echoed in Mill (1874). Our chief concern rests not in philosophical controversies, but rather the everyday psychological understanding of causal assertions, and reasoning from them. The psychological literature is divided on whether the meanings of causal assertions are deterministic or probabilistic. Our aim is to decide between the two accounts.

### Do causes concern possibilities or probabilities?

For many proponents of a deterministic psychological conception of causality, causal claims concern what is possible, and what is impossible (Goldvarg and Johnson-Laird, 2001; Frosch and Johnson-Laird, 2011). The assertion:

Runoff causes contamination to occur.

means that runoff suffices for contamination to occur, though it may occur for other reasons; and the relation is false in case there is runoff without contamination. Hence, the claim can be paraphrased in a conditional assertion that would be false in case its antecedent is true and its consequent is false:

If runoff occurs then contamination occurs.

A categorical assertion such as:

Runoff caused contamination to occur.

can also be paraphrased in a conditional, but one that is counterfactual:

If runoff hadn't occurred then contamination wouldn't have occurred.

The conditional refers to the case in which neither the cause nor its effect occurred. At one time this state was a future possibility, but after the fact it is a possibility that did not occur—it is counterfactual possibility (Johnson-Laird and Byrne, 2002; Byrne, 2005). A more plausible and weaker claim is expressed in a counterfactual conditional allowing that the contamination might have occurred for other reasons:

If runoff hadn't occurred then there mightn't have been contamination.

Not all conditionals express causal relations, so we can ask what else is at stake. One prerequisite is that causes precede their effects, or at least do not occur after them. The two states might be simultaneous in the case of a billiard ball causing a dent in the cushion that it rests on. But, physical contact is not part of the core meaning of a causal relation (cf. Michotte, 1946/1963; Geminiani et al., 1996), because causal assertions can violate it, as in: The moon causes tides. Claims about action at a distance may be false, but their falsity is not merely because they are inconsistent with the meaning of *A causes B*. Likewise, contiguity seems irrelevant to causal assertions about psychological matters, such as: His memory of his behavior embarrassed him.

Many factors—the existence of known mechanisms, causal powers, forces, structures—can be important in inferring a cause (e.g., White, 1995; Ahn and Bailenson, 1996; Koslowski, 1996), and they can be incorporated into the interpretation of a causal assertion or its conditional paraphrase (see Johnson-Laird and Byrne, 1991, for an account of this process, which they refer to as modulation). None of them, however, is part of the core meaning of *A causes B*. Consider mechanistic accounts of causal systems, e.g., how sewing machines work (Miyake, 1986). Experts who use sewing machines can explain their underlying components. However, there comes a point in any such explanation, when everyone must make an assertion equivalent to:

A causes B, and that's that.

This cause has no support. Mechanisms cannot go all the way down—no more than the turtles supporting the earth in primitive cosmology can go all the way down. Hence, mechanisms and their cognates, such as forces and powers, cannot be part of the core meaning of causal assertions.

Granted that causal assertions and their corresponding conditionals concern possibilities, their meaning is deterministic rather than probabilistic. However, some twentieth century theorists, from Russell (1912–1913) to Salsburg (2001, p. 185–6), denied the existence of a coherent notion of causation. Russell was influenced by quantum mechanics, and argued that causation should be replaced by probabilistic considerations. One reason for such skepticism is a failure to divorce beliefs from meanings. Beliefs about causation are often incoherent. For example, some people believe that *it is possible to initiate a causal chain*, and that *every event has a cause*. Both beliefs can't be right, because if every event has a cause, an action to initiate a causal chain has itself a cause, and so it doesn't really initiate the chain. Such beliefs, however, should not be confused with the core meaning of causes, which does not legislate about them: we understand both the preceding assertions that yield the inconsistency. Neither of them seems internally inconsistent.

Other theorists, also inspired by quantum mechanics, have maintained causation but rejected determinism (e.g., Reichenbach, 1956; Suppes, 1970; Salmon, 1980). A cause and its effect are related probabilistically. Reichenbach (1956) argued that a causal assertion, such as:

Runoff causes contamination to occur

means that contamination is more probable given that runoff occurs than given that it does not occur. Hence, a causal claim holds provided that the following relation holds between the two conditional probabilities:

P(contamination | runoff) > P(contamination | no runoff)

The philosophical controversy between determinism and probabilism has spilled over into psychology. Some psychological theories are probabilistic both for causation (e.g., Cheng, 1997, 2000) and for conditionals (Oaksford and Chater, 2007). The case for probabilistic meanings rests in part on causal assertions such as:

Cars cause accidents.

Such assertions tolerate exceptions, which do not refute them, and which therefore imply a probabilistic relation. But, it is the form of the generalization rather than its causal content that enables it to tolerate exceptions. It is a generic assertion akin to:

Cars have radios.

A generic assertion is defined as a generalization with a subject, such as a noun phrase or a gerund, lacking an explicit quantifier (Leslie, 2008). Certain sorts of generic, e.g., *snow storms close schools*, imply a causal connection between their subject, snow storms, and their predicate, close schools. The meaning of the verb, “close,” is causal, and individuals readily infer that snow storms cause an agent to act to close schools (see Prasada et al., 2013). Hence, generics tolerate exceptions. In contrast, if the subjects of assertions contain explicit quantifiers as in:

Some snow storms cause schools to close.

and:

All snow storms cause schools to close.

then the assertions have a deterministic meaning, and the first of these assertions is true as a matter of fact and the second of them is false.

### Evidence against probabilistic accounts of causation

Several arguments count against probabilistic meanings for everyday causal assertions. A major historical problem is to explain why no one proposed such an analysis prior to the formulation of quantum mechanics. Moreover, a singular claim about causation, such as:

The runoff caused contamination to occur

is false if the runoff occurred without contamination. This factual relation is deterministic, and to introduce probabilities into the interpretation of counterfactual conditionals is problematic.

Individuals, as we show later, recognize the difference in meaning between causes and enabling conditions, such as, *The runoff allowed contamination to occur*. But, both increase the conditional probability of an effect given the antecedent, and so the difference in meanings between causes and enabling conditions is impossible to make in probabilistic accounts (Cheng and Novick, 1991; pace Cheng, 2000; Wolff, 2007). The same problem arises in implementing causation in Bayesian networks (Pearl, 2000; Glymour, 2001; Tenenbaum and Griffiths, 2001; Gopnik et al., 2004).

Reasoners often infer a causal relation from a single observation (e.g., Schlottman and Shanks, 1992; White, 1999; Ahn and Kalish, 2000; Sloman, 2005). But, if causal assertions are probabilistic, no single observation could suffice to establish cause and effect, because probabilistic interpretations tolerate exceptions. Lien and Cheng (2000) proposed instead that single observations can refer to previously established causal relations. Repeated observations of billiard balls, for example, establish causal relations about their collisions, which individuals can then use to infer a causal relation from a single new observation. However, as Fair (1979) anticipated, this proposal implies that individuals could never establish causal relations contrary to their expectations.

Interventions that initiate a causal chain are a feature of Bayesian networks (see, e.g., Pearl, 2000; Woodward, 2003), and evidence corroborates their psychological importance (Sloman, 2005; Sloman and Lagnado, 2005). As an example, suppose that the following claim is true:

Overeating causes indigestion.

If we then observe that Max doesn't have indigestion, we can infer that he hasn't overeaten. But, Max could have intervened to prevent indigestion: he could have taken an anti-indigestion pill. In this case, we would no longer make the inference. No special logic or probabilistic considerations are needed to handle these cases (pace Sloman, 2005). Our initial claim is an idealization expressed in a generic, and so it tolerates exceptions.

In summary, the evidence seems to be decisive: causal relations in everyday life have deterministic meanings unless they make explicit reference to probabilities, as in:

Keeping to this diet probably causes you to lose weight.

Moreover, if causation were intrinsically probabilistic, there would be no need for the qualification in this example. Its effect is to weaken the causal claim. Studies of inferences from causal assertions, which we describe below, further bolster their deterministic meanings.

## Mental models of causal assertions

We now turn to the model theory of mental representations, which we outline before we consider its application to reasoning. The theory goes back to Craik (1943) and has still earlier antecedents in philosophy. Its more recent development gives a general account of how individuals understand assertions, how they represent them, and how they reason from them (see, e.g., Johnson-Laird, 1983; Johnson-Laird and Byrne, 1991; Johnson-Laird and Khemlani, 2013). The theory has been implemented computationally, its predictions have been corroborated in psychological experiments and in recent neuroimaging results (e.g., Kroger et al., 2008). And it is of sufficient maturity that given the semantics of a domain such as causation, it calls for few new assumptions in order to account for representation and reasoning.

The first step in understanding an assertion is to parse it in order to construct a representation of its meaning. The theory postulates that the parser's output (an intensional representation) is composed out of the meanings of its parts according to the grammatical relations amongst them. The intensional representation is used to construct, to update, to manipulate, or to interrogate, mental models of the situation under description (an extensional representation). The theory rests on three fundamental principles:

1. Mental models represent *possibilities*: each model captures a distinct set of possibilities to which the current description refers.
2. Mental models are *iconic:* the structure of a model corresponds to the structure of what it represents (see Peirce, 1931–1958, Vol. 4). Hence, kinematic models unfold in time to represent a temporal sequence of events (Khemlani et al., 2013). However, models can also include certain abstract symbols, such as one for negation (Khemlani et al., 2012).
3. The principle of truth: Mental models represent only what is true, not what is false, in each possibility. They yield rapid intuitions. In contrast, *fully explicit* models represent what is false too, but their construction calls for deliberation and access to working memory.

The model theory implements the deterministic meanings of causal relations described in the previous section. An assertion such as:

Runoff causes contamination to occur

has two mental models, one is an explicit model representing the case in which the cause and its effect both occur, and the other is an implicit mental model representing at least one other possibility in which the cause does not occur:

runoff     contamination

             …

The rows in this schematic diagram represent two distinct possibilities. In fact, mental models do not consist of words and phrases, which we use for convenience, but of representations of the objects and events to which the words refer. The ellipsis denotes the other possibilities in which the cause does not occur. These possibilities are not immediately accessible, i.e., one has to work them out. We have omitted from the diagram the temporal relation between cause and effect: the cause cannot come after the effect, and by default comes before it.

The model theory draws a distinction in meaning between causes and enabling conditions (contrary to a tradition going back to Mill, 1874). An enabling condition makes its effect possible: it allows it to happen. The assertion:

Runoff allows contamination to occur.

has a core meaning that is a tautology in which all things are possible provided they are in the correct temporal sequence. Like its corresponding conditional:

If runoff occurs then contamination may occur.

it is possible for runoff to occur, or not to occur, and in either case, with or without contamination. Such assertions are nearly vacuous, and so an obvious implication—an implicature from Grice's (1975) conversational conventions—is that only runoff allows contamination to occur. There are then just three possibilities: with runoff, contamination does or does not occur; but without it, runoff does not occur. The mental models of an enabling assertion are identical to those of a causal assertion. One mental model represents the possibility in which both runoff and contamination occur, and the implicit model represents the other possibilities. A consequence of this identity is that people have difficulty in grasping that causal and enabling assertions differ in meaning. This difficulty has infected the legal systems of both the US and the UK, which make no distinction between the two sorts of causal relation (Johnson-Laird, 1999), though people judge those who cause harmful outcomes as more culpable than those who enable them (Frosch et al., 2007).

When reasoners have to enumerate the possibilities consistent with an assertion, they are able to deliberate and to flesh out their mental models into fully explicit models. The difference between causing and enabling now becomes evident. The fully explicit models of the causal assertion, *runoff causes contamination to occur*, are:

  runoff             contamination

¬runoff             contamination

¬runoff           ¬contamination

where “¬” is a symbol corresponding to a mental token for negation (Khemlani et al., 2012). What the assertion rules out is the possibility that runoff occurs without contamination. In contrast, the fully explicit models of the enabling assertion, *runoff allows contamination to occur*, and its implicature are:

  runoff             contamination

  runoff           ¬contamination

¬runoff           ¬contamination

Some causal claims are stronger than the one above: they assert that the cause is the only way to bring about the effect. The only way to get cholera, for example, is to be infected by the bacterium *Vibrio cholerae*. The corresponding assertion has only two fully explicit models, one in which the cause and effect both occur—the bacterium and the infection, and one in which neither of them occurs. There are also weaker enabling assertions than the one above, that is, ones in which all appropriately temporally-ordered possibilities occur, including the possibility that the effect occurs in the absence of the enabling condition, i.e., the implicature does not occur.

When individuals have to list what is possible, and what is impossible, given each of the main sorts of causal relation, their listings tend to corroborate the model theory (Goldvarg and Johnson-Laird, 2001). Participants list either the three possibilities for *causes* or the two for its stronger interpretation. They are more confused by *enables*, but list the three possibilities above more often than chance, and likewise the four possibilities for its weaker interpretation. They list the three possibilities and the two possibilities for *A prevents B from occurring*, which is synonymous with *A causes B not to occur*.

One attempt to distinguish between causing and enabling in a probabilistic framework is to argue that an enabling condition is constant in the situation, whereas a cause is not (Cheng and Novick, 1991). This difference does occur, but it is not essential according to the model theory. A crucial test used scenarios in which neither the causes nor the enabling conditions were constant (Goldvarg and Johnson-Laird, 2001). Readers may like to try to identify the cause and the enabler in each of the following scenarios:

Given that there is good sunlight, if a certain new fertilizer is used on poor flowers, then they grow remarkably well. However, if there is not good sunlight, poor flowers do not grow well even if the fertilizer is used on them.

and:

Given the use of a certain new fertilizer on poor flowers, if there is good sunlight then the flowers grow remarkably well. However, if the new fertilizer is not used on poor flowers, they do not grow well even if there is good sunlight.

In the first scenario, sunlight is the enabling condition, and the fertilizer is the cause; in the second scenario, the two swap roles. These roles derive from the possibilities to which the respective scenarios refer. In the first scenario, the possibilities are as follows:

  sunlight:           fertilizer          growth

                               ¬fertilizer          growth

                               ¬fertilizer        ¬growth

¬sunlight:                                         ¬growth

As they show, sunlight enables the fertilizer to cause the flowers to grow. Their roles swap in the possibilities for the second scenario. In an experiment, the participants were told that a cause brings about an event whereas an enabling condition makes it possible, and that they had to identify the cause and the enabling condition in sets of scenarios. The order of mention of the cause and enabler was counterbalanced over the scenarios, and each participant saw only one of the four versions of each content. The 20 participants made correct identifications on 85% of the trials, and each of them was right more often than not (Goldvarg and Johnson-Laird, 2001).

These phenomena account against rival accounts of the difference between causes and enabling conditions. The distinction between them is neither capricious nor unsystematic (Mill, 1874; Kuhnmünch and Beller, 2005). It is contrary to the claim that a cause violates a norm assumed by default whereas an enabling condition does not (Einhorn and Hogarth, 1986; Kahneman and Miller, 1986). And the cause need not be conversationally relevant in explanations (Mackie, 1980; Turnbull and Slugoski, 1988; Hilton and Erb, 1996). In sum, the difference in meaning between the two principal sorts of causal assertion is real (see also Wolff and Song, 2003; Sloman et al., 2009).

## Models and causal deductions

How do naïve individuals make causal deductions? One answer is that they rely on the laws of thought, that is, on formal rules of inference akin to those of logic. Indeed, Rips (1994, p. 336) has proposed that formal rules could be extended to deal with causal reasoning. Pure logic makes no reference to specific contents, and so its application to causation depends on the introduction of axioms (or “meaning postulates”), such as:

If A causes B, and B prevents C, then A prevents C

where *A*, *B*, and *C*, are variables that take states or events as their values (von Wright, 1973). Logic, however, has several critical problems in coping with everyday reasoning. One is that infinitely many conclusions follow in logic from any set of premises, and most of them are trivial or silly, such as conjunction of a premise with itself. Another problem is that logic means never having to withdraw the conclusion of a valid inference, even if its conclusion turns out to be false. In jargon, logic is *monotonic*—as you accrue more premises, so you are able to draw more conclusions and never have a warrant for withdrawing any of them. In contrast, everyday reasoning is *nonmonotonic*. You withdraw a conclusion if the facts show it to be wrong.

Another theory is that causal inferences depend on *pragmatic reasoning schemas* (e.g., Cheng et al., 1986). In other words, the axiom above is framed instead as a rule of inference:

A causes B.

B prevents C.

Therefore, A prevents C.

This idea goes back to Kelley's (1973) theory of causal attribution, which postulates such schemas for checking causal relations. Similarly, Morris and Nisbett (1993) proposed a schema including the following two rules:

If cause C is present then effect E occurs.

Cause C is present.

Therefore, Effect E occurs.

and:

If cause C is present then effect E occurs.

Effect E does not occur.

Therefore, Cause C is not present.

In contrast, the model theory makes no use of formal rules of inference, and no use of axioms, meaning postulates, or schemas concerning causation. It simply applies its general principles of reasoning to mental models of causal assertions.

Theorists distinguish among three main sorts of reasoning: deduction, induction, and abduction, which creates hypotheses or explanations. We shall do so too, but with the caveat that human reasoners make inferences without normally concerning themselves about such niceties. To make deductions, individuals draw conclusions that hold in all their models of the premises. To make inductions, they use their knowledge to build models going beyond the information given in the premises, and then infer corresponding conclusions, such as generalizations (Johnson-Laird, 2006). To make abductions, they use their knowledge to incorporate new concepts—those not in the premises—in order to yield causal explanations of everyday events (Johnson-Laird et al., 2004). We will describe the model theory for each of these three sorts of reasoning, starting with deduction here, and we will show that the evidence corroborates its account rather than the alternatives.

At the computational level, the model theory postulates three constraints on everyday reasoning (Johnson-Laird and Byrne, 1991, Ch. 2). First, inferences do not throw away semantic information (see Bar-Hillel and Carnap, 1953). That is, people do not spontaneously make inferences, such as:

Runoff causes contamination.

Therefore, runoff causes contamination or inoculations prevent disease, or both.

The inference is *valid*, because its conclusion must be true if its premise is true. But, its conclusion is less informative (e.g., by a measure of semantic information) than its premise, because the former is compatible with more possibilities than the latter. In contrast, induction and abduction increase semantic information. Second, inferences are parsimonious. For example, a conclusion does not merely consist of a conjunction of all the premises, even though such a conclusion is valid and maintains semantic information. Third, a conclusion should assert something new, and not repeat what is explicit in the premises. If no conclusion meets these three constraints, then individuals respond that nothing follows from the premises—a response that violates logic, but that is perfectly rational. Consider this inference, for instance:

Runoff causes contamination to occur.

Three is a prime number.

What follows?

A logician should respond: infinitely many conclusions, including a conjunction of the first premise with itself 101 times. A more sensible response is: nothing. In short, human reasoners aim not to lose information, to simplify where possible, and to infer something new whether they are making deductive, inductive, or abductive inferences.

The model theory copes with the main sorts of non-monotonicity. It allows for information to be assumed by default, and to be overruled by subsequent information, as when individuals infer that a dog has four legs only to discover that a particular pet is three-legged. It also allows for deductions to be made in an experimental mode ignorant of the facts of the matter, so that when a conclusion turns out to be false, it can be withdrawn without cost. We illustrate such cases in the section below on explanations. It also diverges slightly from logic in its basic assumption about validity. In logic, a valid deduction is one that holds in every case in which the premises hold (Jeffrey, 1981, p. 1). Hence, any conclusion whatsoever follows from inconsistent premises, because there is no case in which the premises hold. The model theory adds a rider for everyday reasoning: there is at least one non-null model in which the premises hold. This proviso blocks valid inferences from inconsistent premises.

At the algorithmic level, the theory postulates that individuals build mental models of premises—they simulate the world under description. They use the information in the premises, their general knowledge, and their knowledge of the context. The system searches for a conclusion that holds in the models and that doesn't merely echo an explicit premise—a principle that holds for conversation in general (Grice, 1975). But, the system can also evaluate given conclusions. A conclusion that holds in all the models of the premises follows of necessity, but if there is a model of the premises in which it does not hold—a counterexample—it does not follow of necessity. Yet, if it holds in most models, it is probable. And if it holds in at least one model, it is possible. Because inferences are based on models of the premises, the resulting conclusions cannot throw semantic information away by adding disjunctive alternatives, or consist of a premise conjoined with itself,

Mental models can be three-dimensional in order to represent spatial relations, and they can be kinematic, unfolding in time to represent a temporal sequence of events (Johnson-Laird, 1983). Evidence supports these hypotheses in the use of mental simulations to deduce the consequences of informal algorithms (Khemlani et al., 2013). Temporal order, however, can also be represented by an axis in a static model.

The “force dynamics” theory of causal reasoning (Barbey and Wolff, 2007; Wolff, 2007) makes analogous claims. It assumes that individuals envisage interacting entities in iconic models in which vectors represent the directions and magnitudes of forces. The theory explains the interpretations of such assertions as:

Pressure will cause the water to remain below 0°C.

Small ridges cause water to stand on the concrete.

The pole will prevent the tent from collapsing.

Each assertion refers to a configuration of forces. The third assertion, for instance, refers to a configuration in which the pole acts against the tendency of the tent to collapse. These tendencies are represented in a vector model. We simplify the diagrams illustrating these models: arrows denote vectors corresponding to the direction and magnitude of forces, and the box denotes the point of stasis, which is the origin of all vectors. The tendency of the tent to collapse is diagramed here, where the two overlaid vectors represent the tent (one vector) heading toward collapse (another vector):

□--->----> collapse

   tent

The pole provides a countervailing force, and so its vector is in the opposite direction:

    <----------□

pole

Because the magnitude of the pole's vector is larger than the magnitude of the tent's vector, the combination of the two yields a small magnitude in the direction away from collapse:

<----□

pole+tent

So, the diagram representing all the interacting vectors is as follows:

    pole+tent

      <-----<---□--->----> collapse

pole               tent

Such diagrams represent a relation in which *A* prevents *B*. Hence, the force theory, like the model theory, postulates that reasoners build up a mental model of causal relations, which can then be scanned to yield inferences. The model theory has not hitherto been formulated to represent forces or the interactions amongst them, and so the force theory contributes an important and hitherto missing component. The resulting models can also underlie kinematic mental simulations of sequences of events.

The model theory can represent probabilities. It uses proportions of models to draw conclusions about *most* entities or *few* of them. These proportions are used to make inferences about probabilities. Individual models can also be tagged with numerals to represent their relative frequencies or probabilities. This algorithmic account unifies deductive and probabilistic reasoning, and it is implemented in an computer program, *mReasoner*, which we continue to develop, and its source code is available at: http://mentalmodels.princeton.edu/models/mreasoner/.

In broad terms, three strands of evidence corroborate the model theory of causal deductions. The first strand of evidence bears out the difference in the possibilities referred to in assertions about causes and assertions about enabling conditions. Readers might like to consider what response they would make to this problem:

Eating protein will cause her to gain weight.

She will eat protein.

Will she gain weight?

Yes, No, and Perhaps yes, perhaps no.

Most participants in an experiment (Goldvarg and Johnson-Laird, 2001) responded: yes. But, when the first premise was instead:

Eating protein will allow her to gain weight

as its fully explicit models predict, the majority rejected the “yes” response. The opposite pattern of results occurred when the second assertion and question were changed to:

She will not gain weight.

Will she not eat protein?

The results therefore bear out the difference in meaning between causing and enabling.

The second strand of evidence supports the deterministic interpretation of causal assertions embodied in the model theory. It rests on the fact that reasoners grasp the force of a counterexample. When they evaluate given inferences, they tend to justify their rejection of an invalid inference by citing a counterexample to its conclusion (Johnson-Laird and Hasson, 2003). Likewise, consider an assertion, such as:

Following this diet causes a person with this sort of metabolism to lose weight.

Participants in experiments were asked about what evidence would refute such claims and similar ones about enabling conditions (Frosch and Johnson-Laird, 2011). In several experiments, every single participant chose a single observation to refute the assertions more often than not, but as the model theory predicts they were more likely to do so for causal assertions than enabling assertions. For both sorts of relation, they chose refutations of the form *A and not-B*, e.g.:

A person with this sort of metabolism followed this diet and yet did not lose weight.

But, as the theory predicts, they chose refutations of the form *not-A and B*, e.g.:

A person with this sort of metabolism did not follow this diet and yet lost weight

more often to refute enabling assertions than causes.

The third strand of evidence concerns the principle of truth and the difference between mental models and fully explicit models. Most of us usually rely on our intuitions, and they are based on a single mental model, which represents only what is true in the corresponding possibility. The following problem illustrates one consequence of this bias:

One of these assertions is true and one of them is false:

      Marrying Evelyn will cause Vivien to relax.

      Not marrying Evelyn will cause Vivien to relax.

The following assertion is definitely true:

      Vivien will marry Evelyn.

Will Vivien relax? Yes/No/It's impossible to know.

The initial rubric is equivalent to an exclusive disjunction between the two causal assertions. It yields the following two mental models:

      Vivien:      marries Evelyn      relaxes

                         ¬marries Evelyn      relaxes

The final categorical assertion eliminates the second possibility, and so most reasoners infer that Vivien will relax. It seems plausible, but the intuition is wrong. The fully explicit models of the disjunction of the two assertions yield only two possibilities, one in which the first assertion is true and the second assertion is false, and one in which the first assertion is false and the second assertion is true. But, in the first case, the second assertion is false, and so Vivien doesn't marry Evelyn and doesn't relax; and, in the second case, the first assertion is false and so Vivien marries Evelyn but doesn't relax. So, the fully explicit and correct models are respectively:

      Vivien:    ¬marries Evelyn    ¬relaxes

                            marries Evelyn    ¬relaxes

The final categorical assertion eliminates the first of them, and it follows that Vivien will not relax. None of the participants in an experiment drew this correct conclusion. The majority inferred that Vivien will relax, and the remainder inferred that it was impossible to know (Goldvarg and Johnson-Laird, 2001).

The model theory makes predictions about causal reasoning that have yet to be tested, though they have been corroborated in other domains. The most important of these predictions are that the more models that have to be taken into account, the more difficult an inference should be, and that a common source of error should be to overlook the model of a possibility. Yet, the evidence we have described here illustrates the case for the model theory, and the alternative theories that we reviewed at the start of this section offer no account of it.

## The induction of causal relations

The vessel, *The Herald of Free Enterprise*, was a roll-on roll-off car ferry. Its bow doors were opened in the harbor to allow cars to drive into the ship, and at its destination, the cars drove off the ship the same way. When it sailed from Zeebrugge in Belgium on March 6th, 1987, the master made the plausible induction about a causal relation, namely, that the assistant bosun had closed the bow doors. The chief officer made the same inference, and so did the bosun. But, the assistant bosun hadn't closed the bow doors: he was asleep in his bunk. Shortly after the ferry left the calm waters of the harbor, the sea poured in through its open doors, and it capsized with the loss of nearly 200 lives. Inductions are risky. There is no guarantee that they yield the truth, and, as this example also illustrates they can concern an individual event, not just generalizations of the sort in textbook definitions of induction.

The risk of inductions arises in part because they go beyond the information in the premises, such as that no-one has reported that the bow doors are open. As a result, they can eliminate possibilities that the premises imply, and they can add relations, such as a temporal order of events within a model of a situation (Johnson-Laird, 1988). In all these cases, the inductive operation depends on knowledge or beliefs. And beliefs are sometimes wrong.

Students of induction from Polya (1973) onwards have postulated formal rules of inference to underlie it—to parallel the formal rules of inference used in logic. These systems have grown ever more sophisticated in programs for machine learning (e.g., Michalski and Wojtusiak, 2007). The model theory, however, assumes that knowledge and beliefs can themselves be represented in models, and so the essential inductive operation is to conjoin two sets of models: one set represents the possibilities derived from the premises, which may be direct observations, and the other set is part of long-term knowledge and beliefs. A simple but common example occurs when knowledge modulates the core interpretation of causality, just as it can do in the interpretation of conditionals (Johnson-Laird and Byrne, 2002). The core meaning of *A causes B*, as we argued earlier, is consistent with three possibilities. Hence, an assertion such as:

A deficiency of some sort causes rickets

refers to three possibilities in which there is a temporal order from cause to effect:

   deficiency    rickets

 ¬deficiency    rickets

 ¬deficiency  ¬rickets

Many people know, however, that rickets has a unique cause –a deficiency in vitamin D, and this knowledge blocks the construction of the second model above in which rickets arise in a person with no deficiency. Modulation in the interpretation of assertions is a bridge from deduction to induction. The resulting models allow one to infer that if a patient has no dietary deficiency, then the patient doesn't have rickets. Logicians can argue that the inference is an enthymeme, that is, it is a valid deduction granted the provision of the missing premise that no other cause for rickets exists. But, one could just as well argue that the inference is an induction, since the conclusion rests on more information than the premises provide. The reasoning system is not concerned with the correct analysis. It relies on whatever relevant knowledge is available to it.

Observations of contingencies can lead to inductive inferences in daily life and in science. Hence, many theories concern inductions from the frequencies of contingencies (e.g., Shanks, 1995; De Houwer and Beckers, 2002; Hattori and Oaksford, 2007; Perales and Shanks, 2007). The analogy with classical conditioning is close. The analyses of frequencies can also yield inductions about causation at one level that feed into those at a higher or more abstract level in a hierarchical Bayesian network (e.g., Gopnik et al., 2004; Griffiths and Tenenbaum, 2005; Lu et al., 2008). Once its structure is established, it can assign values to conditional probabilities that interrelate components in the network, e.g., it can yield the conditional probability of lung cancer given that coughing occurs, and the conditional probability of smoking given lung cancer (see Tenenbaum et al., 2006, for a review).

In contrast, observations can lead to inductions without probabilities. For instance, Kepler analyzed Tycho Brahe's astronomical observations, and used them to induce his three laws of planetary motion, of which the best known is his first law: a planet moves in an elliptical orbit around the sun with the sun at one focus. But, the mind prepared with knowledge can also make an induction from a single observation—a claim supported by considerable evidence (see, e.g., White, 2014). One source of such inferences is knowledge of a potential mechanism, and this knowledge may take the form of a model.

Adults induce new concepts throughout their life. Some are learned from knowledge by acquaintance, others from knowledge by description. You cannot acquire the full concept of a color, a wine, or a sculpture without a direct acquaintance with them, but you can learn about quarks, genes, and the unconscious, from descriptions of them. Likewise, the induction of a generalization is equivalent to the induction of a concept or of a change to a concept, as in Kepler's change to the concept of a planetary orbit. Novel concepts can be put together out of existing concepts. Hence, causal inductions are part of the acquisition of concepts. Causes are more important than effects in the features of a concept. This difference explains why the constituents of natural kinds are important, whereas the functions of artifacts are important (Ahn, 1998). A genetic code is accordingly more critical to being a goat than that it gives milk, whereas that a mirror reflects an image is more important to a mirror than that it is made of glass.

**Figure 1:**
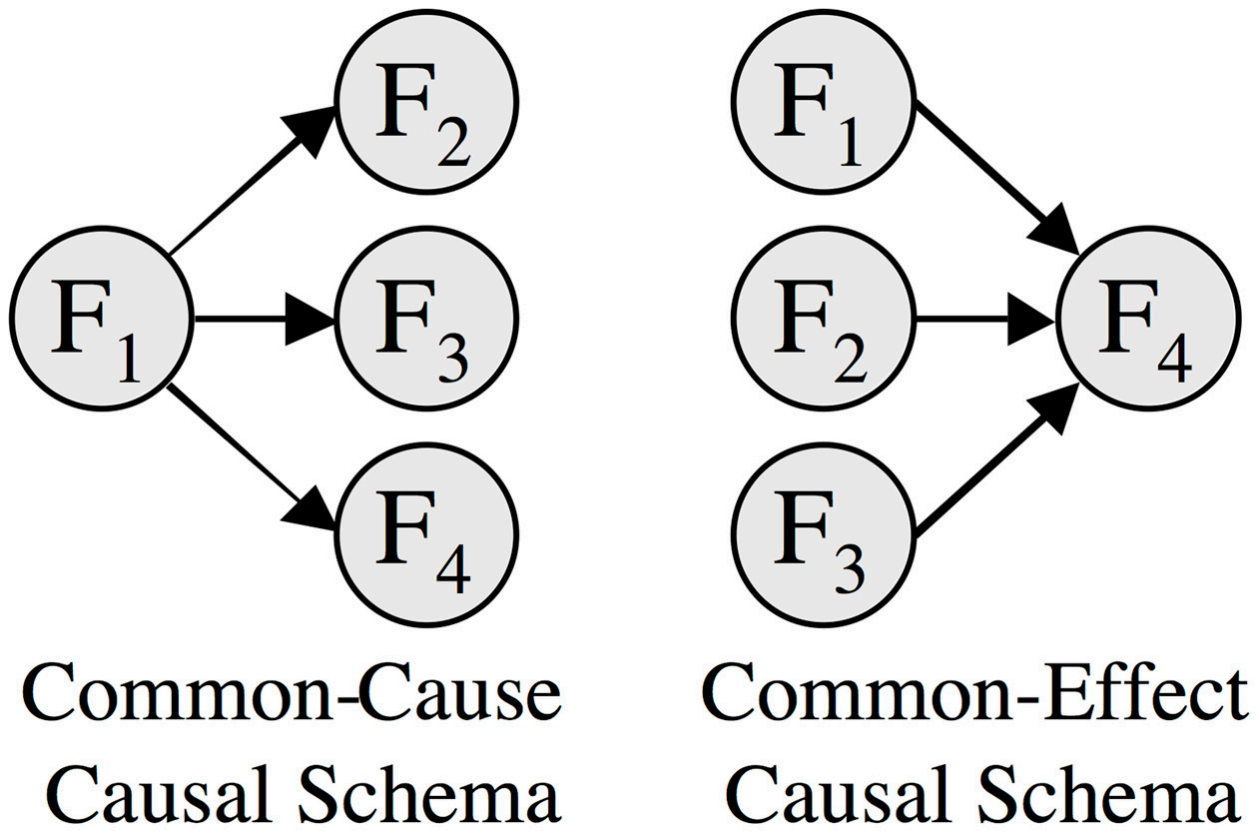
**The common-cause and common-effect causal schemas**. Reproduced with permission from Rehder (2003).

Knowledge of a category's causal structure is important in categorization. Objects are classified as members of a category depending on how well their features fit our intuitive theory, or model, of the causal relations amongst the category's features (see, e.g., Waldmann et al., 1995). Reasoners judge an exemplar as a better instance of a category when its features fit the causal structure of the category (Rehder, 2003). Figure 1 illustrates two contrasting causal structures. In the common-cause structure, one feature is a common cause of three effects, such as the symptoms of a disease, whereas in the common-effect structure, one feature is a common effect of each of three causes, such as a disease that has three independent etiologies. In Rehder's experimental study, which used sensible everyday features, the participants rated category-membership depending on an instance's features, pairs of its features, and high-order relations among its features. The results showed that the participants were indeed sensitive to the difference between the two sorts of causal structure in Figure 1.

At the center of the model theory is the hypothesis that the process of understanding yields a model. In deduction, if a mental model yields a conclusion, its validity can be tested in a search for alternative models. In induction, however, the construction of models increases semantic information. In the case of inductions about specific events in everyday life, this process is part of the normal effort to make sense of the world. Human reasoning relies, wherever possible, on general knowledge. Hence, when the starter won't turn over your car's engine, your immediate inference is that the battery is dead. Another role that knowledge plays is to provide interstitial causal relations that make sense of assertions hitherto lacking them—a process that is case of what Clark (1975) refers to as “bridging” inferences. We demonstrated the potency of such inferences in a series of unpublished studies. One study included a condition in which the participants were presented with sets of assertions for which in theory they could infer a causal chain, such as:

David put a book on the shelf.

The shelf collapsed.

The vase broke.

In another condition, the participants were presented with sets of assertions for which they could not infer a causal chain, such as:

Robert heard a creak in the hall closet.

The faucet dripped.

The lawn sprinklers started.

The theory predicts that individuals should infer the causal relations, and the experiment corroborated this hypothesis. When a further assertion contradicted the first assertion in a set, the consequences were quite different between the two conditions. In the first condition, the contradictory assertion:

David didn't put a book on the shelf

led to a decline in the participants' belief in all the subsequent assertions, and so only 30% of them believed that the vase broke. In the second case, the contradictory assertion:

Robert did not hear a creak in the hall closet

had no effect in the participants' belief in the subsequent assertions. All of them continued to believe that the lawn sprinklers started. This difference in the propagation of doubt is attributable to the causal interpretation of the first sort of scenario, and the impossibility of such an interpretation for the second scenario. This example is close, if not identical, to an abduction, because the attribution of causes explains the sequence of events in the causal scenarios. It leads us to consider abduction in general.

## Abduction of causal explanations

A fundamental aspect of human rationality is the ability to create explanations. Explanations, in turn, depend on understanding: if you don't understanding something, you can't explain it. It is easier to state criteria for what counts as understanding than to define it. If you know what causes something, what results from it, how to intervene to initiate it, how to guide or to govern it, how to predict its occurrence and the course of its events, how it relates to other phenomena, what internal structure is has, how to fix it if it malfunctions, then to some degree you understand it. According to the model theory, “if you understand inflation, a mathematical proof, the way a computer works, DNA or a divorce, then you have a mental representation that serves as a model of an entity in much the same way as, say, a clock functions as a model of the earth's rotation” (Johnson-Laird, 1983, p. 2). And you can use your model to formulate an explanation. Such explanations can help others to understand—to make sense of past events and to anticipate future events. Many psychological investigations have focused on explanatory reasoning in the context of specific, applied domains, such as fault diagnosis (e.g., Besnard and Bastien-Toniazzo, 1999) and medical decision-making (e.g., Ramoni et al., 1992). But, as Hume (1748/1988) remarks in the epigraph to this paper, most reasoning about factual matters is founded on cause and effect. To illustrate the role of models in causal abductions, consider this problem:

If someone pulled the trigger, then the gun fired.

Someone pulled the trigger, but the gun did not fire.

Why not?

Most people presented with the problem offered a causal explanation, such as:

Someone unloaded the gun and so there were no bullets in it.

They even rated such an explanation as more probable than either the cause alone or the effect alone (Johnson-Laird et al., 2004). In daily life, explanations tend to explain only what needs to be explained (Khemlani et al., 2011), but, as the case above illustrates, causal relations take priority over parsimony (pace Lombrozo, 2007). In science, Occam's razor calls for parsimonious explanations.

When the preceding problem is couched in these terms:

If someone pulled the trigger, then the gun fired.

The gun did not fire.

Why not?

many individuals preferred a causal explanation to a simple deductive one:

No one pulled the trigger.

The bias does not appear to reflect cultural background, and it is much the same for Westerners and East Asians (Lee and Johnson-Laird, 2006), but it is sensitive to personality. Individuals who are, or who feel, open to experience and not so conscientious tend to make the causal explanation, whereas their polar opposites tend to make the deductive explanation (Fumero et al., 2010).

The nonmonotonic retraction of a conclusion and modification of beliefs is a side effect of explanation. When individuals explain what's going on in a scenario, they then find it harder to detect an inconsistency it contains than when they have not formulated an explanation (Khemlani and Johnson-Laird, 2012). Conversely, they are faster to revise assertions to make them consistent when they have explained the inconsistency first (Khemlani and Johnson-Laird, 2013). And they rate explanations as more plausible and probable than modifications to the premises that remove the inconsistency—a pattern of judgments that occurs both in adults (Khemlani and Johnson-Laird, 2011) and in children (Legare, 2012). In short, the priority in coping with inconsistencies is to find a causal explanation that resolves them. Explanations first, nonmonotonic modifications after.

## The lateral prefrontal cortex and mental models for causal inference

A critical brain region underlying mental models for causal inference is the lateral prefrontal cortex, which is known to encode causal representations and to embody the three foundational assumptions of the model theory (see the earlier account of the theory): mental models represent possibilities; their structure can be iconic, mimicking the structure of what they represent; and they represent what is true at the expense of what is false. We now turn to a review of the neuroscience evidence linking each assumption of these principles to core functions of lateral prefrontal cortex.

### Mental models represent possibilities

The lateral prefrontal cortex is known to play a central role in the representation of behavior-guiding principles that support goal-directed thought and action (Miller and Cohen, 2001). Such top-down representations convey information about possible states of the world, representing what goals are available in the current environment and what actions can be performed to achieve them.

The lateral prefrontal cortex represents causal relations in the form of learned task contingencies (causal relations, which neuroscientists refer to as if-then rules). Asaad and colleagues trained monkeys to associate each of two cue objects (*A* and *B*) with a saccade to the right or a saccade to the left (Asaad et al., 1998). The authors found relatively few lateral prefrontal cortex neurons whose activity simply reflected a cue (e.g., *A*) or response (e.g., a saccade to the right). Instead, the modal group of neurons (44% of the population) showed activity that reflected the current association between a visual cue and the directional saccade it instructed. For example, a given cell might be strongly activated only when object *A* instructed “saccade left” and not when object *B* instructed the same saccade or when object *A* instructed another saccade. Likewise, lateral prefrontal cortex neurons acquire selectivity for features to which they are initially insensitive but that are behaviorally important. For example, Watanabe trained monkeys to recognize that certain visual and auditory stimuli signaled whether or not a reward, a drop of juice, would be delivered (Watanabe, 1990, 1992). He found that neurons in the lateral prefrontal cortex came to reflect specific cue-reward dependencies. For example, a given neuron could show strong activation to one of the two auditory (and none of the visual) cues, but only when it signaled reward.

Studies of monkeys and humans with lateral prefrontal cortex damage also suggest that this region is critical for representing causal principles (if-then rules) that underlie goal-directed thought and adaptive behavior. Early studies of the effects of prefrontal cortex damage in humans suggested its role in goal-directed behavior (e.g., Ferrier, 1876) and since then broad consensus in the literature implicates this region in the ability to control lower-level sensory, memory, and motor operations in the service of a common goal (Shallice, 1982; Duncan, 1986; Passingham, 1993; Grafman, 1994; Wise, 1996). Contemporary lesion mapping studies in large populations of patients with focal brain damage further indicate that selective damage to the lateral prefrontal cortex produces impairments in the ability to acquire and use behavior-guiding rules (causal relations) that are central to higher cognitive functions, including general intelligence (Barbey et al., 2012b), fluid intelligence (Barbey et al., 2012a, 2014a), cognitive flexibility (Barbey et al., 2013), working memory (Barbey et al., 2011), and discourse comprehension (Barbey et al., 2014b). In monkeys, damage to ventrolateral prefrontal cortex also impairs the ability to learn causal relations in tasks (Petrides, 1982, 1985; Halsband and Passingham, 1985; Murray et al., 2000). Most, if not all, tasks that are disrupted following prefrontal cortex damage rely on mental models that capture the causal structure of experience (cf. Passingham, 1993).

Further evidence implicating the lateral prefrontal cortex in causal inference is provided by the fMRI literature (for reviews, see Barbey and Patterson, 2011; Patterson and Barbey, 2012). An important study by Satpute and colleagues demonstrates activity within the dorsolateral prefrontal cortex for the processing of causal vs. associative relations (Satpute et al., 2005). Selective activity within the dorsolateral prefrontal cortex for causal (rather than associative) inference provides evidence against associationist accounts of causal representation and instead supports the mental models framework.

In sum, the reviewed findings indicate that the lateral prefrontal cortex represents causal relations that establish mappings between possible states of the world, providing the links that bind situations, actions and consequences necessary for goal-directed behavior. These mappings are believed to bias competition in other parts of the brain responsible for task performance (Miller and Cohen, 2001). Thus, signals in the lateral prefrontal cortex guide activity along pathways that connect task-relevant sensory inputs, memories, and motor outputs, which can be naturally represented in the form of mental models of causal relations.

### Mental models are iconic

The information processing architecture of the lateral prefrontal cortex supports the iconic nature of mental models: the structure of a model corresponds to the structure of what it represents in the visual, spatial, auditory, motor and kinematic domains. The cytoarchitectonic areas that comprise lateral prefrontal cortex are often grouped into three regional subdivisions that emphasize processing of particular information based on their interconnections with specific cortical sites. Ventrolateral prefrontal cortex is heavily interconnected with cortical regions for processing information about visual form and stimulus identity (inferior temporal cortex), supporting the categorization of environmental stimuli in the service of goal-directed behavior. Dorsolateral prefrontal cortex is interconnected with cortical areas for processing auditory, visuospatial, and motor information (parietal cortex), enabling the regulation and control of responses to environmental stimuli. Finally, anterolateral prefrontal cortex is indirectly connected (via the ventromedial prefrontal cortex) with limbic structures that process internal information, such as emotion, memory and reward (Goldman-Rakic, 1995; Fuster, 2008; Petrides et al., 2012). The lateral prefrontal cortex is therefore connected with virtually all sensory neocortical and motor systems and a wide range of subcortical structures, supporting the iconic nature of mental models in the visual, spatial, auditory, motor, and kinematic domains. The lateral prefrontal cortex integrates information across this broadly distributed set of systems and is known to support higher-order symbolic representations, such as negation (Tettamanti et al., 2008), that go beyond modality-specific knowledge (Ramnani and Owen, 2004).

### Mental models represent only what is true

A third property of lateral prefrontal cortex function is that it represents directly experienced (i.e., “true”) events and actively maintains these representations over time in a highly accessible form (i.e., storage of information via sustained neuronal activity patterns). The capacity to support sustained activity in the face of interference is a distinguishing feature of the lateral prefrontal cortex. Sustained neural activity within the lateral prefrontal cortex was first reported by Fuster (1973), who demonstrated that neurons within the lateral prefrontal cortex remain active during the delay between a presented cue and the later execution of a contingent response. Such sustained neural activity often represents a particular type of information, such as the experienced location or identity of a stimulus (Fuster and Alexander, 1971; Kubota and Niki, 1971; Fuster, 1973; Funahashi et al., 1989; di Pellegrino and Wise, 1991) or a particular relation between a stimulus and its corresponding response (Asaad et al., 1998).

### Summary

In summary, mental models for causal inference critically depend on the lateral prefrontal cortex, and neuroscience evidence indicates that this region extracts goal-relevant features of experience (causal relations or if-then rules), it can construct iconic representations, and they represent only what is true.

## General discussion

In Ibsen's play, Dr. Stockmann sought to prevent further contamination of the public bath facility by alerting the town to the problem. To *prevent* an outcome is to cause it not to occur, and so he acted in the hope that his causes would have consequences. The meaning of a causal relation according to the model theory concerns possibilities: a cause suffices to bring about the effect, which does not precede the cause; an enabling condition makes such an effect possible; and a preventative action causes the effect not to occur. We have argued that reasoners interpret causal assertions by simulating the situation, i.e., by building a mental model, to which the assertions refer, and then they inspect that model to draw conclusions from it. Their initial mental models reflect intuitive interpretations of causal relations, e.g., their initial model of *runoff causes contamination to occur* is identical to that of *runoff enables contamination to occur*, i.e.:

runoff      contamination

               …

The first row of the diagram represents a possibility in which runoff occurs concurrently with contamination, and the second row of the diagram represents that other possibilities are consistent with the assertion. The theory therefore explains why reasoners often conflate causes and enabling conditions, i.e., the mental models of the assertions are the same. When prompted to deliberate about alternative possibilities, however, reasoners are able to flesh out the models and can distinguish causes from enabling conditions (Goldvarg and Johnson-Laird, 2001).

The model theory is deterministic. It posits that causal assertions are used to build discrete models of possibilities. The construction of these discrete models excludes continuous probabilistic information. Three overarching phenomena support a deterministic interpretation of causality:

- reasoners can infer causal relations from single observations;
- they distinguish causes from enabling conditions
- they refute causal assertions with single instances.

None of these effects is consistent with a probabilistic interpretation of causality.

Reasoners make deductions, inductions, and abductions from causal premises. They base their causal deductions on mental models of the premises; they infer conclusions from the possibilities corresponding to those of the premises. Models can include information about the dynamics of forces. The evidence corroborating the model theory shows that individuals succumb to fallacies—illusory inferences—because mental models do not represent what is false in a possibility (Goldvarg and Johnson-Laird, 2001). Causal induction depends on the use of background knowledge to build models that go beyond the information in the premises. And causal abduction is a complex process in which knowledge is used to introduce new causal relations, which are not part of the premises, in order to provide explanations. Explanation takes priority over the nonmonotonic retraction of conclusions and the editing of propositions to eliminate inconsistencies.

The evidence from neuroscience strongly implicates lateral prefrontal cortex as the site of causal processing, and corroborates the principal assumptions of the theory. Just as there are untested behavioral claims of the theory, so too many aspects of causal processing in the brain have yet to be investigated. Inferences from causal assertions, for example, should yield a time course reflecting the successive activation of linguistic areas and then prefrontal activation—a time course that has been observed in studies of deduction in other domains (Kroger et al., 2008). Similarly, materials that elicit visual imagery as opposed to spatial representations impede reasoning, because they elicit irrelevant activity in visual cortex (Knauff et al., 2003). Analogous effects may also occur in causal reasoning. Likewise, recent evidence to support the hierarchical organization of lateral prefrontal cortex function may reflect the complexity of causal representations for goal-directed thought and behavior (for reviews, see Ramnani and Owen, 2004; Badre, 2008).

In sum, the model theory provides a comprehensive account of causal reasoning: what causal assertions mean, how they are interpreted to build models, how these models underlie deductive conclusions; how they incorporate background information in inductive inferences and abductive explanations.

### Conflict of interest statement

The authors declare that the research was conducted in the absence of any commercial or financial relationships that could be construed as a potential conflict of interest.
